# Complete mitochondrial genome and phylogenetic analysis of *Sinocyclocheilus microphthalmus* (Cypriniformes: Cyprinidae)

**DOI:** 10.1080/23802359.2019.1667274

**Published:** 2019-09-19

**Authors:** Peng Li, Jian Yang

**Affiliations:** Key Laboratory of Environment Change and Resources Use in Beibu Gulf, Nanning Normal University, Nanning, China

**Keywords:** Mitochondrial genome, *Sinocyclocheilus microphthalmus*, phylogeny

## Abstract

*Sinocyclocheilus microphthalmus* is endemic to Youjiang River and Hongshuihe River systems in Guangxi, Southwestern China. In this study, the complete mitochondrial genome of *S. microphthalmus* was sequenced. It was determined to be 16,589 bases. The overall base composition was 31.87% A, 24.96% T, 27.19% C, 15.99% G with 43.17% GC content. The nucleotide sequence data of 12 heavy-strand protein-coding genes of *S. microphthalmus* and other 13 *Sinocyclocheilus* species were used for phylogenetic analyses. Trees constructed using Bayesian inference showed a topology demonstrating that *Sinocyclocheilus* species clustered as one monophyletic clade with strong supports.

*Sinocyclocheilus microphthalmus*, an endemic cyprinid fish in China, was first found in the Chengbihe River (belong to Youjiang River) of Lingyun county and distributed in the underground stream of the Youjiang River and Hongshuihe River systems in Guangxi, Southwestern China (Li 1989; Lan et al. 2013). In this study, the complete mitochondrial genome of *S. microphthalmus* was generated, then phylogenetic analysis of genus *Sinocyclocheilus* was performed using protein-coding genes in the mitochondrial genome. It would be useful for genetics and evolutionary studies on this species in the future.

The sample of *S. microphthalmus* was collected from Haokun Lake (belong to Chengbihe River) in Lingyun county of Guangxi, China (24°11′53.95″N, 106°41′5.31″E). The voucher specimen was preserved in 95% ethanol and deposited in the Zoological Specimen Museum of Nanning Normal University, with an accession number NNNU201712001. Total genomic DNA was extracted using standard phenol/chloroform methods (Sambrook et al. 1989). The DNA library was constructed and sequenced using Illumina Hiseq 4000 platform (Illumina, San Diego, CA, USA). The complete mitochondrial genome was assembled with SOAPdenovo v2.04 (Luo et al. 2012) and MITObim v1.6 (Christoph et al. 2013). The assembled genome was annotated using the DOGMA (Wyman et al. 2004) and then submitted into the GenBank database with accession number MN145877.

The complete mitochondrial genome of *S. microphthalmus* was 16,589 bp in length, which consists of 13 protein-coding genes, 22 tRNA genes, and 2 rRNA genes, and a D-loop locus (control region). The overall base composition was 31.87% A, 24.96% T, 27.19% C, 15.99% G with 43.17% GC content. The ND6 and 8 of the 22 tRNAs genes (tRNA^Gln^, tRNA^Ala^, tRNA^Asn^, tRNA^Cys^, tRNA^Tyr^, tRNA^Ser^(UCN), tRNA^Glu^, tRNA^Pro^) were encoded on the light strand and the others were encoded on the heavy strand. COI gene started with GTG codon, while the other 12 protein-coding genes started with ATG. Nine of protein-coding genes ended with complete stop codon (TAA or TAG), while 4 genes (COII, ND3, ND4, and CYTB) used incomplete stop codon (T–).

To present phylogenetic relationships in the genus *Sinocyclocheilu*s, phylogenetic trees were derived from the concatenated sequence of 12 protein-coding genes from 14 *Sinocyclocheilus* species and 2 outgroup species (*Cyprinus cario* and *Danio rerio*) by employing Bayesian inference. Partitioned Bayesian phylogenetic analyses were conducted using MrBayes 3.1.2 (Ronquist and Huelsenbeck 2003). The best-fitting model (GTR + I + G) of sequence evolution for Bayesian analyses was obtained by Modeltest 3.7 (Posada and Crandall 1998) under the Akaike Information Criterion.

The phylogenetic result was similar to the previous molecular evidence (Figure 1) (Li et al. 2017, 2018). It showed that all *Sinocyclocheilus* species clustered as one monophyletic group and grouped into two major clades with strong supports. *Sinocyclocheilus microphthalmus* and 12 species formed one clade, while *S. jii* formed the other.

**Figure 1.**
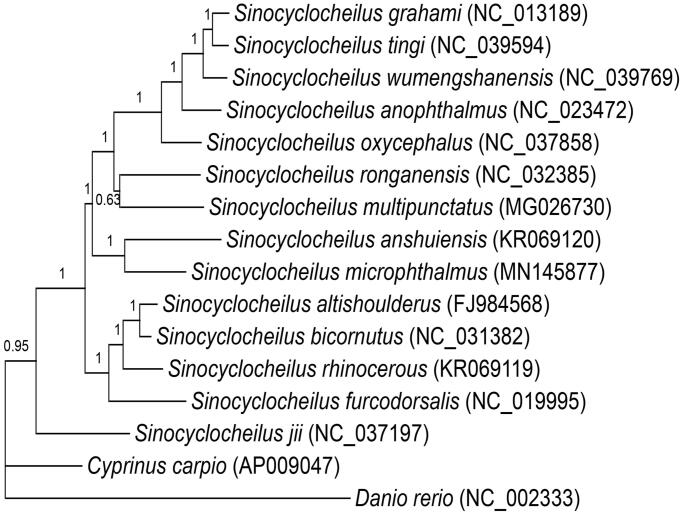
Bayesian 50% majority-rule consensus phylogenetic tree of *S. microphthalmus. Cyprinus cario* and *Danio rerio* were used as outgroups. Numbers on the internode branches are Bayesian posterior probability.
